# Evaluation of Anticarcinogenic and Cytotoxic Effects of COX-2 Specific Inhibitors on an Animal Model of Hepatocellular Carcinoma Using Isolated Mitochondria

**DOI:** 10.5812/ijpr-164947

**Published:** 2025-08-30

**Authors:** Alireza Mahmudi, Enayatollah Seydi, Nahid Ahmadi, Zhaleh Mohsenifar, Mahsa Azami Movahed, Afshin Zarghi, Jalal Pourahmad

**Affiliations:** 1 School of Pharmacy, Shahid Beheshti University of Medical Sciences, Tehran, Iran; 2 Department of Occupational Health and Safety Engineering, School of Health, Alborz University of Medical Sciences, Karaj, Iran; 3 Research Center for Health, Safety and Environment, Alborz University of Medical Sciences, Karaj, Iran; 4 Department of Pharmaceutical Chemistry, School of Pharmacy, Semnan University of Medical Sciences, Semnan, Iran; 5 Department of Pharmaceutical Chemistry, School of Pharmacy, Shahid Beheshti University of Medical Sciences, Tehran, Iran; 6 Department of Pathology, School of Medicine, Shahid Beheshti University of Medical Sciences, Tehran, Iran; 7 Department of Toxicology and Pharmacology, School of Pharmacy, Shahid Beheshti University of Medical Sciences, Tehran, Iran

**Keywords:** Hepatocellular Carcinoma, COX-2 Inhibitors, Mitochondria, Animal Model

## Abstract

**Background:**

Cancer is regarded as one of the most significant health concerns in the world. Hepatocellular carcinoma (HCC) is a malignancy with high incidence and mortality rates and can lead to death. Cyclooxygenase (COX)-2 is responsible for the development of various cancers, including HCC. Therefore, the use of COX-2 inhibitors can help in the prevention and treatment of cancer.

**Objectives:**

The present study aimed to synthesize and examine the effect of imidazolium [1,2-a] piperidinium (4cl-A) and benzo [d] imidazo [1,2-b] thiazolium (1-naphtyl-C) compounds as COX-2 inhibitors on the rat model of HCC.

**Methods:**

Animals were randomly assigned to control and HCC induction groups. The study duration was 15 weeks. The HCC was induced using DEN (200 mg/kg, ip) at a single dose and 2-AAF (dietary, 0.02% w/w, for 2 weeks). After 15 weeks, the investigation focused on mitochondrial toxicity parameters. One-way and two-way ANOVA statistical tests were used to analyze the data.

**Results:**

The results showed that 4cl-A and 1-naphtyl-C can reduce mitochondrial activity, increase the level of free radicals (ROS), collapse in mitochondrial membrane potential (MMP), cause swelling of mitochondria, and release cytochrome c from HCC mitochondria. While this effect was not observed in healthy mitochondria.

**Conclusions:**

The results of the study indicate that these COX-2 inhibitors, along with selected drugs, can help in the treatment of HCC. However, more clinical studies should be conducted.

## 1. Background

Cancer is recognized as one of the world’s major health problems, with the incidence of various cancers on the rise. Estimates project that the yearly death toll from cancer will reach nearly 21 million by 2030 (1, 2). Hepatocellular carcinoma (HCC) is the most common and significant type of liver cancer, and it is both deadly and dangerous. Hepatocellular carcinoma is a global health concern, and its incidence rate is increasing (3-5). Known risk factors for HCC include hepatitis B and C viruses, environmental carcinogens, excessive alcohol consumption, and fatty liver disease. Despite different treatment methods, their effectiveness in managing HCC remains a significant challenge (3, 6, 7). Consequently, there is a pressing need for new therapeutic approaches with different mechanisms of action to improve treatment outcomes for this cancer.

Cyclooxygenase (COX)-2 is recognized as a factor involved in the process of carcinogenesis and cancer progression in humans. The COX-2 has been shown to increase proliferation, cell viability, angiogenesis, and cell invasion while inhibiting apoptosis and suppressing the immune system (8-10). Although it is not easily detected in most tissues under normal conditions, it is strongly induced in abnormal conditions and due to inflammatory stimuli (11). Overexpression of COX-2 causes inflammation, which is a crucial stimulus for liver fibrosis induction (12). The relationship between high COX-2 levels and the tumorigenesis and progression of HCC has been demonstrated in previous studies. Also, it has been reported that HCC patients with high COX-2 expression have a poor prognosis. Therefore, its inhibition can help in the prevention and treatment of cancer.

Our previous research has shown that COX-2 inhibitors can lead to an increase in the level of reactive oxygen species (ROS) in cancer mitochondria (13, 14). The electron transport chain (ETC) in mitochondria is the primary source of free radical (especially ROS) production (15, 16). The role of ROS in HCC tumorigenesis is double-edged. An excessive increase of ROS causes cytotoxic effects on tumor cells, which can eventually induce cell death through oxidative stress. Accordingly, regulating the level of ROS in various cancer cells can be a suitable approach for cancer treatment (17-19). Nowadays, targeted molecular therapy has been investigated by researchers as a treatment approach for HCC. It has been established that certain targeted drugs cause cell death in HCC cells by raising the level of ROS (20). Therefore, the use of COX-2 inhibitor compounds that can increase the level of ROS in HCC cells may help in the treatment of this cancer along with selected drugs.

## 2. Objectives

The present study aimed to examine the effect of imidazolium [1,2-a] piperidinium (4cl-A) and benzo [d] imidazo [1,2-b] thiazolium (1-naphtyl-C) compounds as COX-2 inhibitors on the rat model of HCC.

## 3. Methods

### 3.1. Animals

The purchased animals (male Wistar rats) were kept under standard laboratory conditions, including controlled temperature, humidity, and lighting cycle. The ethics and animal supervision committee’s guidelines were followed in all experiments (IR.SBMU.PHARMACY.REC.1401.253). This study aimed to cause minimal suffering to the animals.

### 3.2. Chemistry

**Figure 1.**
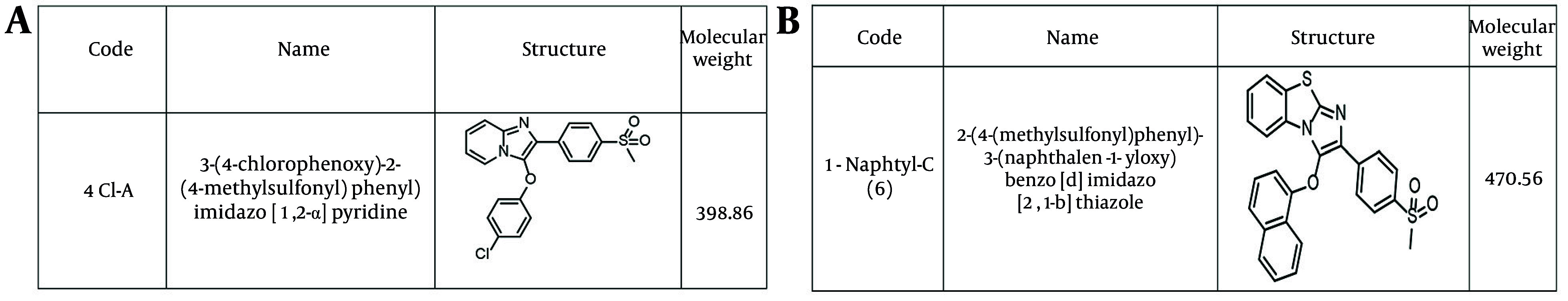
A, the specifications of the 4cl-A; B, the specifications of the 1-naphtyl-C.

**Figure 2.**
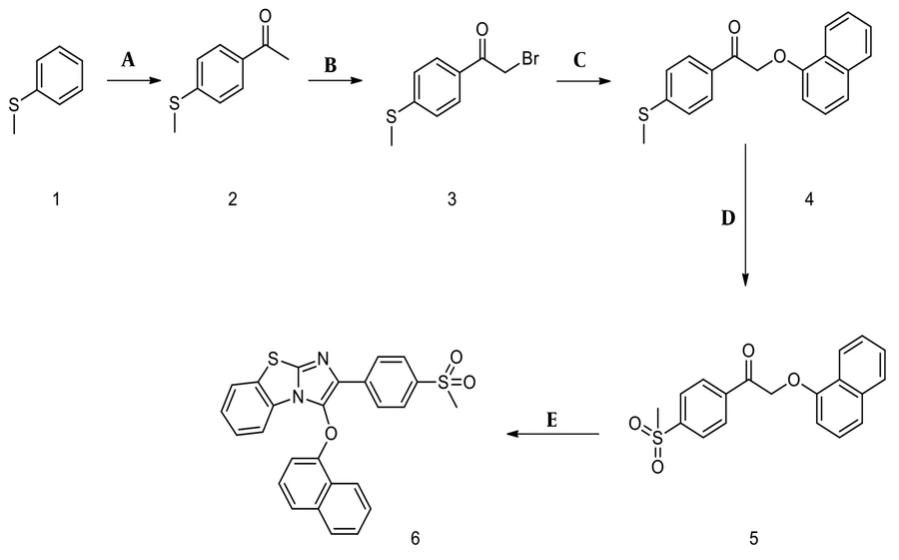
Synthesis of benzo[d]imidazo[2,1-b]thiazole derivative. Reagents and conditions: A, AlCl_3_, CH_3_COCl, CHCl_3_, 25°C, and 2h; B, Br_2_, CH_3_OH, and RT; C, K_2_CO_3_, 1-naphthol, acetone, reflux, and 5h; D, Oxone, THF, water, RT, and 5h; E, I2 (2eq), 2-aminobenzothiazole (3eq), DMF, 120°C, and 24 h.

The specifications of the compounds are shown in Figure 1A and B. The target compound 6 was synthesized in five steps, as outlined in Figure 2. In the first step, thioanisole was acetylated to 4-(methylthio)acetophenone 2 using the Friedel-Crafts acylation reaction. Then, the 4-(methylthio)acetophenone 2 afforded the bromoacetyl derivative 3 by α-bromination reaction. Compound 3 was treated with 1-naphthol to give intermediate 4, which was oxidized by Oxone and yielded the corresponding compound 5. These steps were performed based on the previous study (21). Finally, the cyclization of intermediate 5 was carried out using elemental iodine and 2-aminobenzothiazole to obtain the desired final product 6 (22). The purity of the final compound was checked with TLC using various solvents with different polarities. The chemical structure was characterized by FTIR, ^1^H NMR, ^13^C NMR, and ESI-MS.

#### 3.2.1. Procedure for the Synthesis of Compound 6

One mmol of compound 5 was dissolved in 15 mL of DMF. Then, 3 mmol of 2-aminobenzothiazole and 2 mmol of iodine were added, and the reaction mixture was refluxed for 24 hours. After the completion of the reaction (monitored by TLC), a saturated sodium thiosulfate solution (1 mL) was added to the reaction mixture and stirred until the brown color disappeared. The mixture was then added to a beaker containing ice. The resulting precipitate was filtered, dried, and recrystallized using ethanol to give the final product, 2-(4-(methylsulfonyl)phenyl)-3-(naphthalen-1-yloxy)benzo[d]imidazo[2,1-b]thiazole.

Yield, 78%; white crystalline powder; melting point: °C; IR (KBr disk): ν (cm^-1^) 1144, 1304 (SO_2_); ^1^H NMR (DMSO-d₆): δ ppm 3.21 (s, ^3^H, SO_2_Me), 6.91 (d, ^1^H, J = 7.68 Hz, naphthyloxy H_2_), 7.24 (d, ^1^H, J = 8.0 Hz, imidazobenzothiazole H_5_), 7.31 - 7.38 (m, ^2^H, imidazobenzothiazole H_6_ - H_7_), 7.43 (t, ^1^H, naphthyloxy H_3_), 7.791 - 7.933 (m, ^5^H, naphthyloxy H_4_ and H_6_ - H_7_ and methylsulfonylphenyl H_2_ and H_6_), 8.086 - 8.165 (m, ^4^H, naphthyloxy H_5_ and methylsulfonylphenyl H_3_ and H_5_ and imidazobenzothiazole H_8_), 8.68 (d, ^1^H, J = 8.2 Hz, naphthyloxy H_8_); ^13^C NMR (DMSO-d₆): δ ppm 43.94, 109.98, 113.37, 117.03, 125.60, 125.65, 125.73, 126.03, 127.28, 127.57, 127.73, 128.15, 128.25, 129.78, 129.98, 130.59, 131.27, 131.62, 134.25, 135.04, 137.67, 139.17, 143.39, 154.27; LC-MS (ESI): m/z: 471 (M+1).

### 3.3. Experimental Design

The animals were divided into two groups for HCC induction after a week of habituation. Group 1 was considered the control group. Group 2 was considered the HCC induction group, receiving DEN (200 mg/kg, ip) and 2-AAF (dietary, 0.02% w/w). The study was conducted for 15 weeks. Biochemical and pathological tests were carried out after the 15th week to determine HCC induction (data not reported) (23). In the next step, mitochondria were incubated with different concentrations of COX-2 inhibitors (4cl-A and 1-naphtyl-C), and then toxicity tests were conducted.

### 3.4. Isolation of Mitochondria from Rat Hepatocytes

Ketamine (80 mg/kg, intraperitoneal) and xylazine (5 mg/kg, intraperitoneal) were administered to the animals at the end of week 15. The first step was to isolate hepatocytes using a standard protocol (24, 25). To isolate the mitochondria, the hepatocytes were centrifuged twice: First at 760 × g for 5 minutes, and then at 8000 × g for 20 minutes (26, 27). Toxicity parameters were evaluated after mitochondria were incubated with different concentrations of COX inhibitors (4cl-A and 1-naphtyl-C). In this study, mitochondrial health and integrity were assessed through the MTT test (for evaluation of mitochondrial function/mitochondrial complex II) and cytochrome c oxidase (complex IV) assay kit, respectively.

### 3.5. Evaluation of Succinate Dehydrogenase Activity

Measurement of mitochondrial evaluation of succinate dehydrogenase (SDH) activity was done using the MTT test. Each test was conducted using 1 mg protein/mL of mitochondria. To determine this parameter, mitochondria were suspended in the appropriate assay buffer and then incubated with COX inhibitors (4cl-A and 1-naphtyl-C) for 1 hour. The activity of mitochondrial SDH in both groups was measured by evaluating absorbance at a wavelength of 570 nm (28).

### 3.6. Evaluation of Reactive Oxygen Species Assay

The DCFH-DA probe was used to measure the ROS level by suspending mitochondria in a respiration buffer assay. Next, mitochondria were incubated with different concentrations of 4cl-A (10, 20, and 40 µg/mL) and 1-naphtyl-C (5, 10, and 20 µg/mL). Lastly, the assayed fluorescence intensity level (EX = 488 nm/EM = 527 nm) indicates the level of mitochondrial ROS generation. At 15, 30, and 60 minutes following incubation with different concentrations of COX inhibitors (4cl-A and 1-naphtyl-C), the ROS levels in both groups were evaluated.

### 3.7. Evaluation of Mitochondrial Membrane Potential Assay

The Rh123 probe was used to measure mitochondrial membrane potential (MMP) collapse by suspending mitochondria in a corresponding assay buffer. Next, mitochondria were incubated with different concentrations of 4cl-A (10, 20, and 40 µg/mL) and 1-naphtyl-C (5, 10, and 20 µg/mL). Lastly, the assayed fluorescence intensity level (EX = 490 nm/EM = 535 nm) indicates the MMP collapse. At 15, 30, and 60 minutes following incubation with different concentrations of COX inhibitors (4cl-A and 1-naphtyl-C), the MMP collapse in both groups was evaluated.

### 3.8. Evaluation of Mitochondrial Swelling

The initial step was to suspend mitochondria in the appropriate assay buffer. Then, mitochondria were incubated with several concentrations of 4cl-A (10, 20, and 40 µg/mL) and 1-naphtyl-C (5, 10, and 20 µg/mL). The absorbance of each sample was measured at a wavelength of 540 nm. The test was conducted at 15, 30, and 60 minutes following the incubation of 4cl-A (10, 20, and 40 µg/mL) and 1-naphtyl-C (5, 10, and 20 µg/mL).

### 3.9. Measurement of Cytochrome c Release

After mitochondria incubation with 4cl-A (20 µg/mL) and 1-naphtyl-C (10 µg/mL), cytochrome c release and the effects of inhibitory compounds were evaluated according to the manufacturer’s kit instructions.

### 3.10. Statistical Analysis

The report of results was based on the mean ± SD. Data analysis was carried out using GraphPad Prism software (version 8). The level of significance was set at P < 0.05. A one-way ANOVA test was used for the assessment of SDH activity and cytochrome c release. Furthermore, a two-way ANOVA test was used for the assessment of ROS generation, MMP collapse, and mitochondrial swelling.

## 4. Results

### 4.1. Effects of 4cl-A and 1-naphtyl-C on Mitochondrial Evaluation of Succinate Dehydrogenase Activity

**Figure 3.**
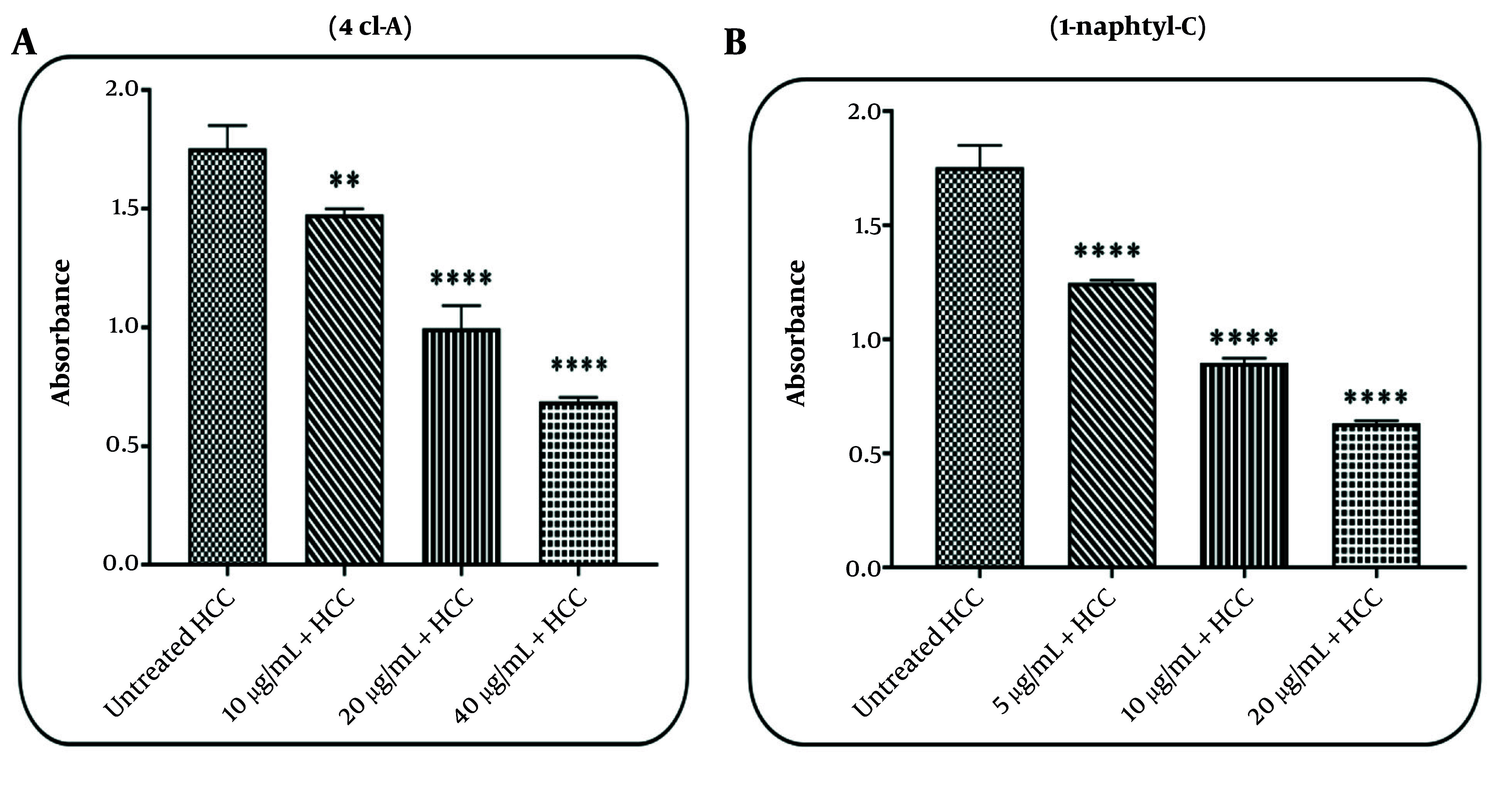
Evaluation of succinate dehydrogenase (SDH) activity: The effect of A, 4cl-A (10, 20, and 40 µg/mL) and B, 1-naphtyl-C (5, 10, and 20 µg/mL) on mitochondrial SDH activity [data were represented as mean ± SD; n = 3; ** P < 0.01 and **** P < 0.0001 significant difference with untreated hepatocellular carcinoma (HCC) group].

In the HCC group, results revealed that the activity of mitochondrial SDH was decreased by 4cl-A (10, 20, and 40 µg/mL; Figure 3A) and 1-naphtyl-C (5, 10, and 20 µg/mL; Figure 3B). These two compounds have no effect on mitochondrial SDH activity in healthy mitochondria (the data are not shown). These results suggest that mitochondrial function in the HCC group can be decreased by these two COX inhibitor compounds.

### 4.2. Effects of 4cl-A and 1-naphtyl-C on Mitochondrial Reactive Oxygen Species

**Figure 4.**
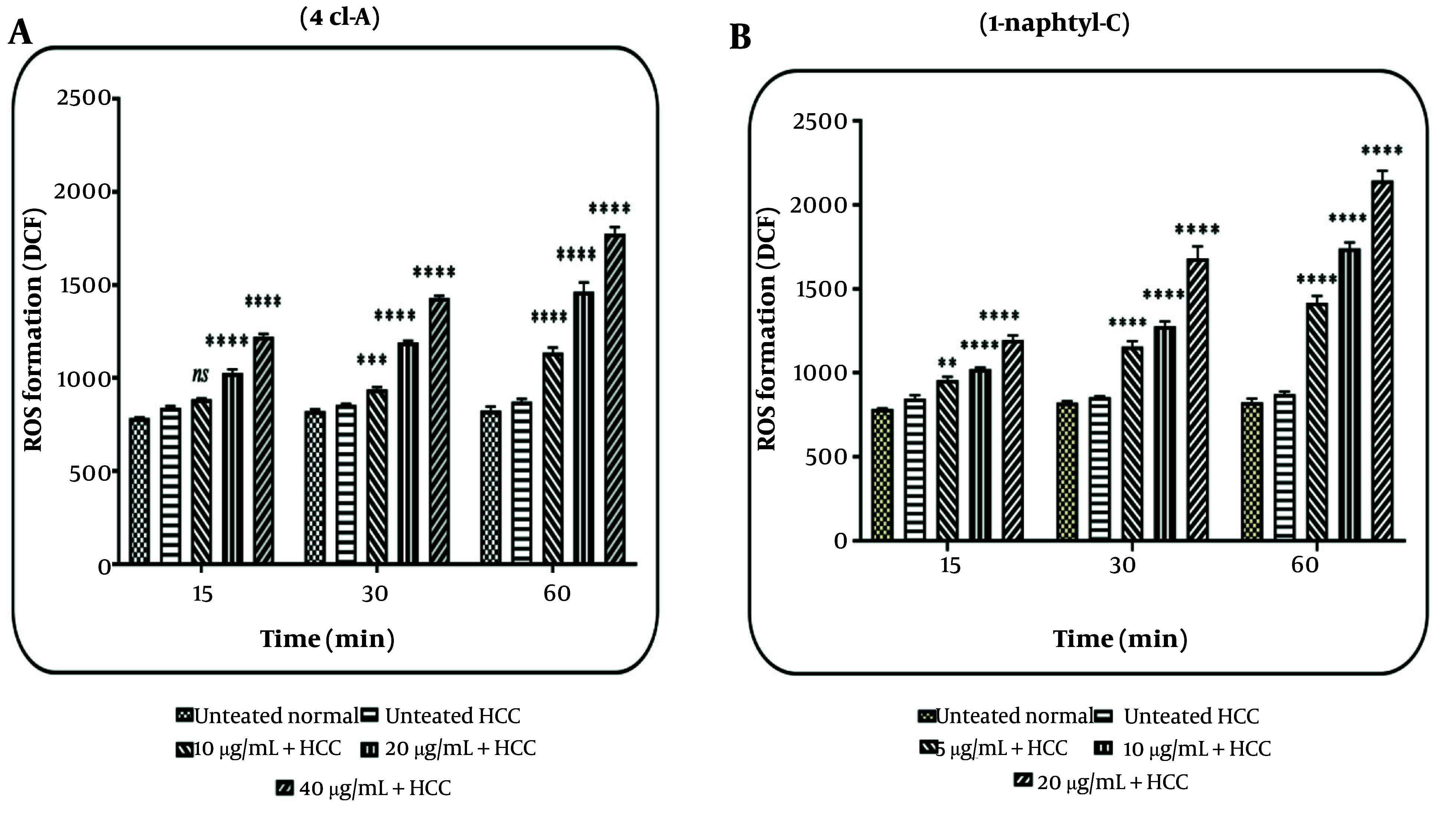
Reactive oxygen species (ROS) assay: The effect of A, 4cl-A (10, 20, and 40 µg/mL) and B, 1-naphtyl-C (5, 10, and 20 µg/mL) on mitochondrial ROS [data were represented as mean ± SD; n = 3; ** P < 0.01, *** P < 0.001, and **** P < 0.0001 significant difference with untreated hepatocellular carcinoma (HCC) group].

The results revealed that 4cl-A (10, 20, and 40 µg/mL) and 1-naphtyl-C (5, 10, and 20 µg/mL) have considerably increased the ROS level in the HCC group (Figure 4A and B). This effect was not observed in the normal group (the data are not shown). An increase in ROS level was observed at 15, 30, and 60 minutes after incubation of mitochondria with 4cl-A (10, 20, and 40 µg/mL; Figure 4A) and 1-naphtyl-C (5, 10, and 20 µg/mL; Figure 4B). 

### 4.3. Effects of 4cl-A and 1-naphtyl-C on Mitochondrial Membrane Potential Collapse

**Figure 5.**
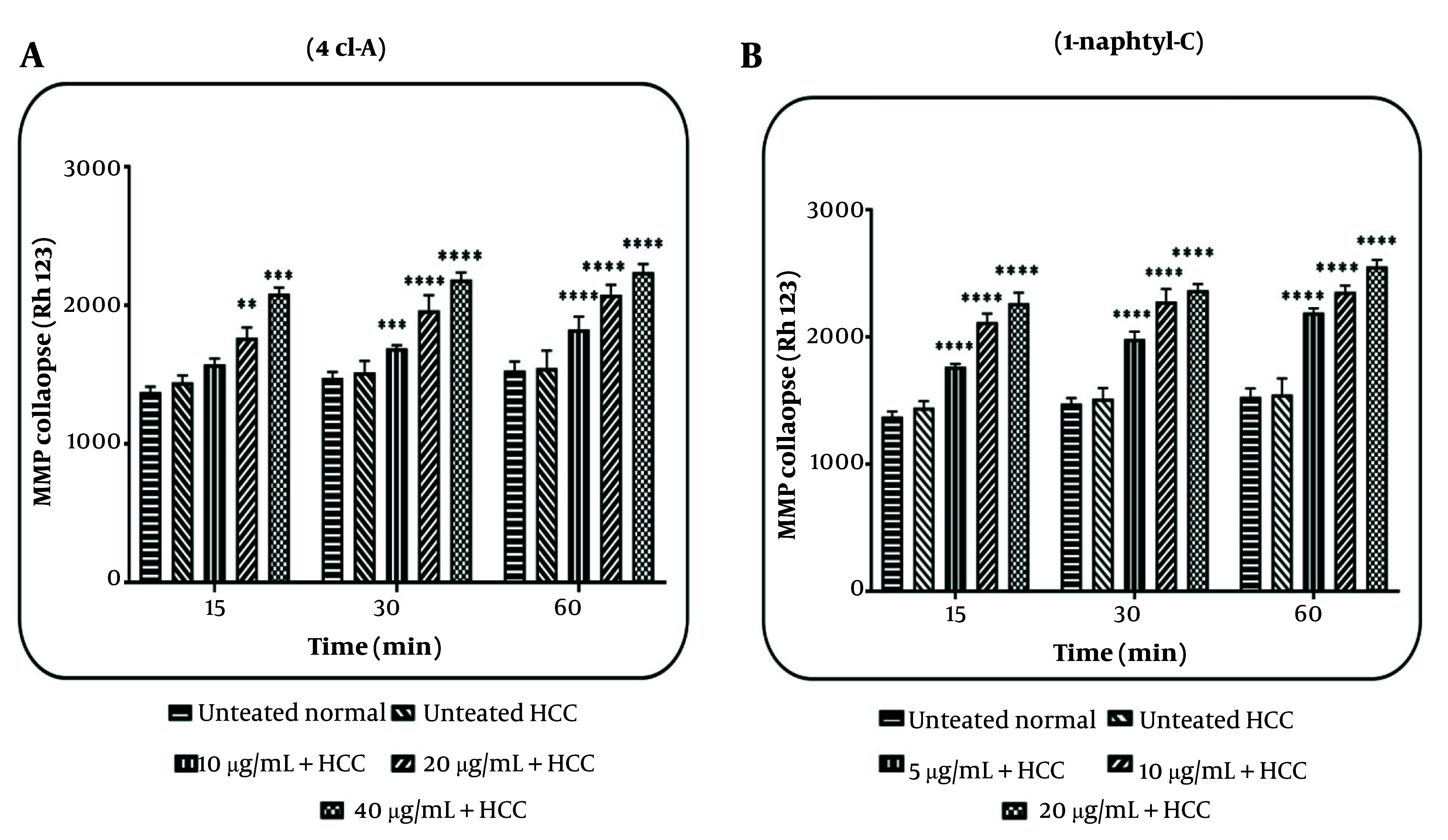
Mitochondrial membrane potential (MMP) collapse assay: The effect of A, 4cl-A (10, 20, and 40 µg/mL) and B, 1-naphtyl-C (5, 10, and 20 µg/mL) on MMP collapse [data were represented as mean ± SD; n = 3; ** P < 0.01, *** P < 0.001, and **** P < 0.0001 significant difference with untreated hepatocellular carcinoma (HCC) group].

After incubation of mitochondria for 15, 30, and 60 minutes with 4cl-A (10, 20, and 40 µg/mL; Figure 5A) and 1-naphtyl-C (5, 10, and 20 µg/mL; Figure 5B), the results indicate a collapse in the MMP in the HCC group. The release of pro-apoptotic proteins can be a consequence of a collapse in the MMP.

### 4.4. Effects of 4cl-A and 1-naphtyl-C on Mitochondrial Swelling

**Figure 6.**
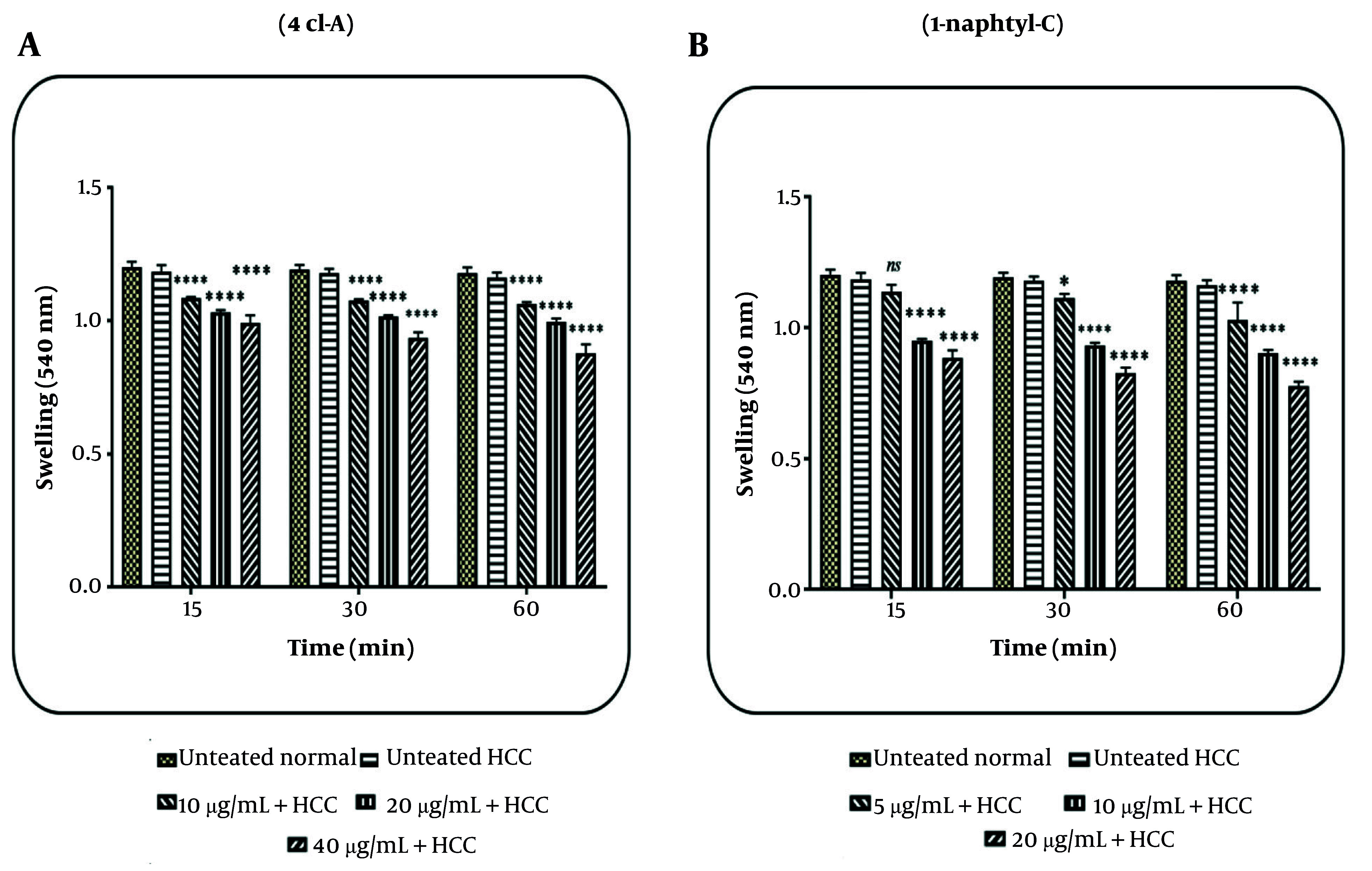
Mitochondrial swelling assay: The effect of A, 4cl-A (10, 20, and 40 µg/mL) and B, 1-naphtyl-C (5, 10, and 20 µg/mL) on mitochondrial swelling [data were represented as mean ± SD; n = 3; * P < 0.05, and **** P < 0.0001 significant difference with untreated hepatocellular carcinoma (HCC) group].

In the HCC group, results revealed that mitochondrial swelling was increased by 4cl-A (10, 20, and 40 µg/mL; Figure 6A) and 1-naphtyl-C (5, 10, and 20 µg/mL; Figure 6B). In the normal group, these two compounds do not affect mitochondrial swelling (the data are not shown). The results indicate that these compounds can cause damage to mitochondria.

### 4.5. Effects of 4cl-A and 1-naphtyl-C on Cytochrome c Release

**Figure 7.**
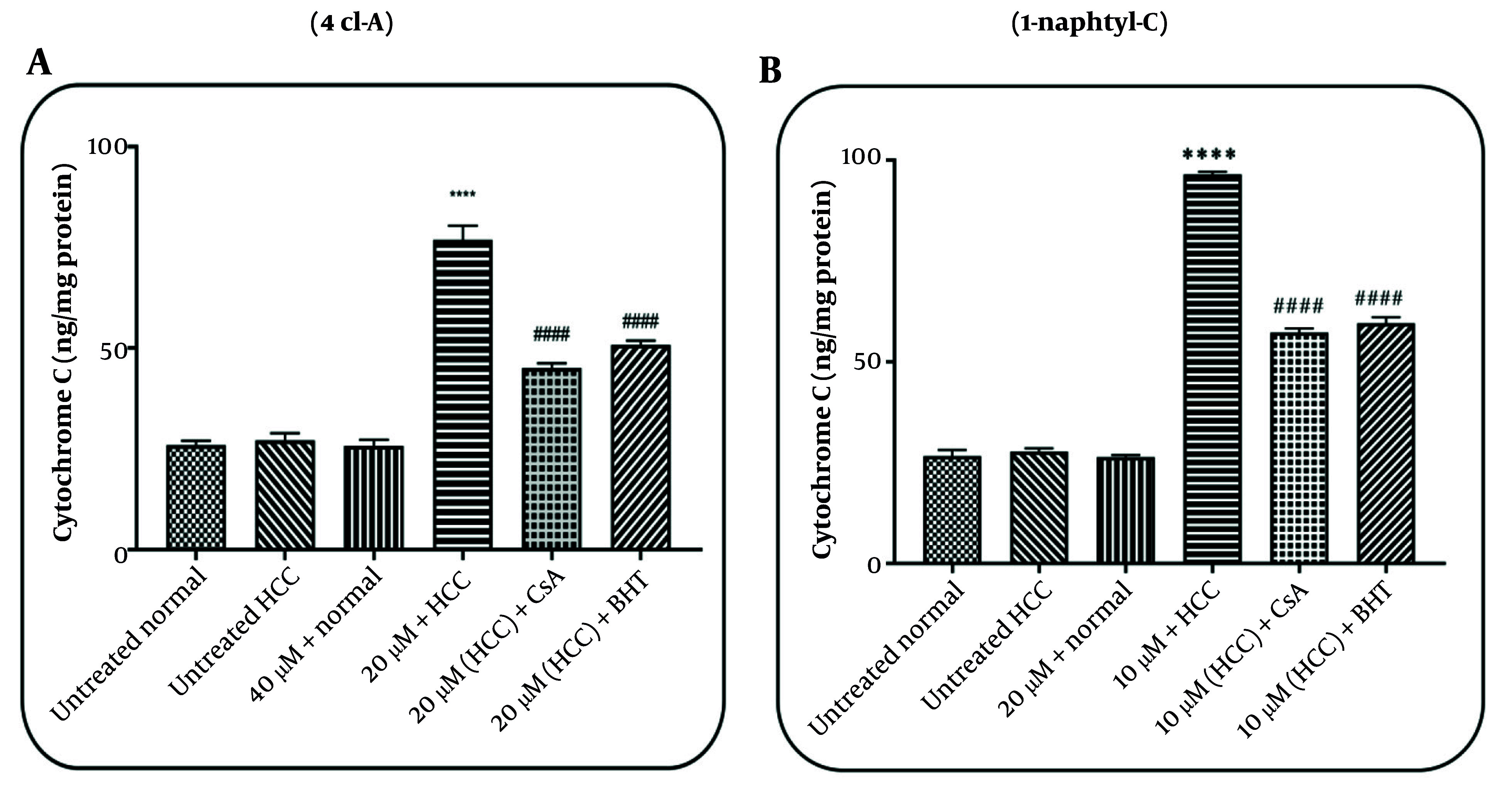
Cytochrome c release assay: The effect of A, 4cl-A (20µg/mL) and B, 1-naphtyl-C (10 µg/mL) on cytochrome c release [data were represented as mean ± SD; n = 3; **** P < 0.0001 significant difference with untreated hepatocellular carcinoma (HCC) group; #### P < 0.0001 significant difference with 20 µg/mL + HCC group].

After incubation of mitochondria with 4cl-A (20 µg/mL; Figure 7A) and 1-naphtyl-C (10 µg/mL; Figure 7B), the results indicate a release of cytochrome c in the HCC group. The release of cytochrome c was not reported after the incubation of mitochondria with a high concentration of 4cl-A (40 µg/mL; Figure 7A) and 1-naphtyl-C (20 µg/mL; Figure 7B) in the normal group. Furthermore, the results indicated that CsA and BHT, as inhibitors, decrease the effect of 4cl-A (20 µg/mL; Figure 7A) and 1-naphtyl-C (10 µg/mL; Figure 7B) on the release of cytochrome c from HCC mitochondria (Figure 7A and B).

## 5. Discussion

Our research focused on investigating the mechanism of the effect of COX-2 inhibitors on mitochondria from an HCC rat model, with the objective of aiding HCC treatment. In recent years, many researchers have investigated mitochondria as an important target for the treatment of various cancers. Additionally, scientists are focused on compounds that have the ability to target mitochondria in cancer cells (29-31). Mitochondria are one of the most important intracellular organelles that play crucial roles in various physiological conditions, and the effect of compounds on them can be associated with significant changes in the cell (32-34). The investigation focused on the effects of COX inhibitors (4cl-A and 1-naphtyl-C) on mitochondrial parameters that could be responsible for cell death.

According to research, COX-2 is involved in both carcinogenesis and the proliferation and progression of cancer cells. Additionally, COX-2 exhibits the ability to suppress apoptosis and stimulate angiogenesis in cancer cells (10, 11, 35). Liver cancer has been shown to have high levels and expression of COX-2, which could lead to tumorigenesis in these patients (8, 35-37). Inhibition of COX-2 can aid in the prevention and treatment of this cancer. Our previous studies have demonstrated that compounds that inhibit COX-2 can kill cancer cells through their effect on mitochondria (13, 14). Our initial findings showed that 4cl-A and 1-naphtyl-C were able to affect mitochondrial SDH activity and decrease its activity. The results indicate that these COX-2 inhibitors can cause dysfunction of cancer mitochondria.

Mitochondria are known as important producers of ROS (38, 39). The ROS play significant roles at different levels in cells. Accordingly, ROS has been investigated as an important target in cancer treatment by researchers. The ROS at low levels can play a role in the process of carcinogenesis, but at high and toxic levels, they can help kill cancer cells and aid in the treatment of cancer. Furthermore, ROS at high levels can induce apoptosis and have anti-tumor effects (16-19, 40). It has also been shown that these oxidative agents can play a role in killing HCC cells. One of the approaches of targeted drugs has been the creation of ROS in HCC cells, leading to the induction of apoptosis in these cells (5, 20, 41). Therefore, the use of compounds that can increase the level of ROS in cancer cells may be an important approach in cancer treatment. The results of our study showed that 4cl-A and 1-naphtyl-C can increase the level of ROS in HCC mitochondria. It is possible that COX-2 inhibitors in this study caused the production of ROS in HCC mitochondria through their effect on the mitochondrial ETC.

Further, our results showed that 4cl-A and 1-naphtyl-C, as COX inhibitors, caused mitochondrial swelling, collapse in MMP, and release of cytochrome c in the HCC mitochondria. These events may be caused by the generation of ROS in HCC mitochondria. Researchers have shown that ROS can cause the opening of the mitochondrial permeability transition pore (MPTP), which results in mitochondrial swelling, collapse in the MMP, and the release of pro-apoptotic compounds (42-44). Therefore, 4cl-A and 1-naphtyl-C may affect the mitochondrial ETC, causing the generation of ROS, which can lead to further events. In this study, it was shown that the use of an antioxidant (BHT) and an MPT pore inhibitor (CsA) decreased the effects of COX-2 inhibitors.

### 5.1. Conclusions

In conclusion, we suggest a novel effect mechanism of 4cl-A and 1-naphtyl-C involving an increase in ROS generation, leading to mitochondrial swelling, MMP collapse, and subsequent cytochrome c release. These results suggest that mitochondria can be a target for COX-2 inhibitory compounds and provide a theoretical approach for its clinical application in HCC treatment.

## Data Availability

The datasets generated and/or analyzed during the current study are available from the corresponding authors on reasonable request.
